# A Survey on Computational Methods for Investigation on ncRNA-Disease Association through the Mode of Action Perspective

**DOI:** 10.3390/ijms231911498

**Published:** 2022-09-29

**Authors:** Dongmin Bang, Jeonghyeon Gu, Joonhyeong Park, Dabin Jeong, Bonil Koo, Jungseob Yi, Jihye Shin, Inuk Jung, Sun Kim, Sunho Lee

**Affiliations:** 1 Interdisciplinary Program in Bioinformatics, Seoul National University, Seoul 08826, Korea; 2 Interdisciplinary Program in Artificial Intelligence, Seoul National University, Seoul 08826, Korea; 3 Department of Computer Science and Engineering, Seoul National University, Seoul 08826, Korea; 4 Department of Computer Science and Engineering, Kyungpook National University, Daegu 41566, Korea; 5 MOGAM Institute for Biomedical Research, Yongin-si 16924, Korea; 6 AIGENDRUG Co., Ltd., Seoul 08826, Korea

**Keywords:** non-coding RNA, disease association, network mining, deep learning, mode of action, integrative analysis

## Abstract

Molecular and sequencing technologies have been successfully used in decoding biological mechanisms of various diseases. As revealed by many novel discoveries, the role of non-coding RNAs (ncRNAs) in understanding disease mechanisms is becoming increasingly important. Since ncRNAs primarily act as regulators of transcription, associating ncRNAs with diseases involves multiple inference steps. Leveraging the fast-accumulating high-throughput screening results, a number of computational models predicting ncRNA-disease associations have been developed. These tools suggest novel disease-related biomarkers or therapeutic targetable ncRNAs, contributing to the realization of precision medicine. In this survey, we first introduce the biological roles of different ncRNAs and summarize the databases containing ncRNA-disease associations. Then, we suggest a new trend in recent computational prediction of ncRNA-disease association, which is the *mode of action (MoA) network* perspective. This perspective includes integrating ncRNAs with mRNA, pathway and phenotype information. In the next section, we describe computational methodologies widely used in this research domain. Existing computational studies are then summarized in terms of their coverage of the MoA network. Lastly, we discuss the potential applications and future roles of the MoA network in terms of integrating biological mechanisms for ncRNA-disease associations.

## 1. Introduction

Proteins are the most useful biomarkers for diseases and also the most effective therapeutic targets. However, the Human Genome Project revealed that about 98% of the genome does not encode proteins. Meanwhile, an intriguing phenomenon of RNA-mediated inhibition of protein synthesis was observed in the early 1990s [1]. These unknown transcripts that are not translated into protein are called noncoding RNAs (ncRNAs), and are now considered as regulatory mediators of the biological system.

NcRNAs play important roles in various molecular mechanisms, such as RNA editing, silencing, gene activation, and protein translation [2]. Some of them are known as biomarkers of a certain disease and/or as a drug target. Additionally, ncRNA expression profile can be used as a drug response predictor [3]. In the pharmaceutical industry, various ncRNA-based therapeutics are undergoing clinical trials [4].

The academic interest of each ncRNA varies, depending on its time of discovery, sequencing technology, and so on. NcRNAs discovered so far include microRNAs (miRNAs), long non-coding RNAs (lncRNAs), circular RNAs (circRNAs), small interfering RNAs (siRNAs) and piwi-interacting RNAs (piRNAs).

In this review, we focus on the computational studies for discovering association between diseases and ncRNAs, such as miRNAs, lncRNAs, and circRNAs. First, we describe each type of ncRNAs and its association with diseases. Databases containing ncRNA-disease associations are also summarized. Then, we propose a novel perspective called *the Mode of Action (MoA) network*, a heterogeneous network-integrative approach inspired by the pharmaceutical domain. We also provide a brief overview of the computational methodologies used in various studies, grouped into network mining and network learning. The following sections are at the heart of this review, summarizing the computational models on non-coding RNA-disease associations (ncDAs) through the MoA network perspective, and also show how this integrative approach is becoming a new trend. After reviewing the studies based on their coverage of the MoA network, the future role of the MoA network in computational ncRNA-disease association prediction is discussed.

## 2. Types of ncRNAs and Its Association with Diseases

### 2.1. miRNA

First discovered in 1993 from *C. elegans*, miRNA is a small single-stranded ncRNA molecule with a length of about 21–24 nucleotides [5]. At the molecular level, miRNAs interact with complementary mRNA molecules and regulate the expression level of target genes. Important biological processes affected by miRNA include cell development [6], apoptosis [7], inflammation [8] and DNA damage response [9].

Since circulating miRNAs are stable and easily detectable, they have been used as biomarkers for some diseases [10]. For example, patients with myocardial infarction showed high expression of miR01, miR-133, miR-208 and miR-499 [11,12,13], which control cardiac conductance by regulating action potential or expression of sarcomeric contractile proteins. Recent studies reported that miR-29a and miR-29b contribute to the pathogenesis of diabetes mellitus by regulating insulin signaling pathways [14].

### 2.2. lncRNA

lncRNA is a ncRNA molecule with a length of more than 200 nucleotides. Since the discovery of the first lncRNAs in the early 1990s, studies have investigated the functions of lncRNAs. LncRNA is now known as a crucial regulatory component that acts as a decoy, scaffold, miRNA sponge and so on [15]. Biological processes affected by various lncRNAs include dosage compensation, genomic imprinting, or cell differentiation [16].

Increasing evidences support the association between lncRNA and disease. For instance, *BACE1* was reported as an essential factor for the production of the toxic amyloid precursor protein, which serves a major role in Alzheimer’s disease [17]. Additionally, a recent study revealed that the expression level of *HAND2-AS1* is high in liver cancer stem cells. It recruits a chromatin-remodeling complex, resulting in the activation of BMP signaling to promote self-renewal of cancer cells [18].

### 2.3. circRNA

In the 1970s, circular forms of linear single-stranded RNA molecules were reported in several RNA viruses, which was the first discovery of circRNA [19]. CircRNA is a continuous loop that is covalently closed and has one to five exons, which are usually generated by back-splicing. This RNA molecule acts mainly as a miRNA sponge and deactivates miRNA’s mechanisms by binding with the seed regions of miRNA [20]. It also participates in other RNA–protein interactions and protein translations by forming a complex with proteins. Biological processes affected by circRNA include cell survival, proliferation [21] and the TGF-β signaling pathway [22].

Unlike linear RNAs, circRNAs lack free ends, which makes them 2.5 times more stable than their linear counterparts [23]. Owing to its stability and abundance, circRNAs are regarded as potential circulating biomarkers for diseases diagnosis or drug response [24]. It has been reported that circRNA_0025202 regulates the miR-182-5p/FOXO3a axis in breast cancer, resulting in tamoxifen sensitivity and tumor progression [25]. Additionally, circHIPK3 has been reported as miRNA a sponge that affects the viability, migration, and proliferation of retinal endothelial cells [21].

### 2.4. The ceRNA Hypothesis

Competing endogenous RNA (ceRNA), first hypothesized by Salmena et al. [26] in 2011, refers to the network of ncRNAs that regulate each other through competition for their targets, e.g., miRNAs and mRNAs. The ceRNA hypothesis was based on the observation of the regulating effect of *PTENP1* pseudogene on tumor suppressor gene *PTEN* 3’UTR region, leading to growth inhibition in a DICER-dependent manner [27]. Since the regulatory interaction between lncRNA and miRNA has been elucidated, evidences showing different types of ncRNA regulation have been accumulated.

With the increasing evidence of ncRNAs’ regulatory interactions, miRNA, lncRNA and circRNAs, along with mRNAs have now become the core elements of the ceRNA network. The ceRNA network is now regarded as a critical component in understanding the association between transcriptome and disease occurrence [28,29,30].

## 3. Databases

There are several databases that describe experimental evidence of ncDAs. Some provide the differentially expressed ncRNA lists in certain diseases, and others also offer ncDA expression profiles. We summarize those databases in Table 1, annotated with the major types of ncRNAs and a short description of each database.

## 4. The Mode of Action Network for ncRNA-Disease Association

A majority of ncDA studies try to understand or infer their relationships directly, without considering biological mechanisms of ncRNA. However, the effect of ncRNA has to pass through multiple entities at intermediate levels before resulting in clinical outcome. A desirable approach would be interpreting the effect of ncRNA at various levels, starting with mRNA expression level then observing how its alteration leads to disease onset through perturbation in biological pathway and cellular phenotype. This perspective is consistent with the results of recent studies which reported performance improvement in clinical outcome prediction by integrating multiple biological entities, compared to the ones using only a single data type [43,44,45,46,47].

This approach is similar to the investigation on the effect of administered chemicals as mode of action (MoA) of chemicals [48,49]. In order to investigate the relationship between chemical and disease, changes in various levels of biological organization, e.g., gene expression, pathway and phenotypes, are examined. Going beyond from *describing* known drug-disease treatment pairs, several computational tools have been proposed to *predict* novel drug-disease associations by examining the pattern of known MoAs, forming a research field now widely known as drug repositioning [50,51]. Reflecting the MoA concept through integration of biological entities of different levels is now a new trend in computational prediction of drug-disease association.

**Figure 1:**
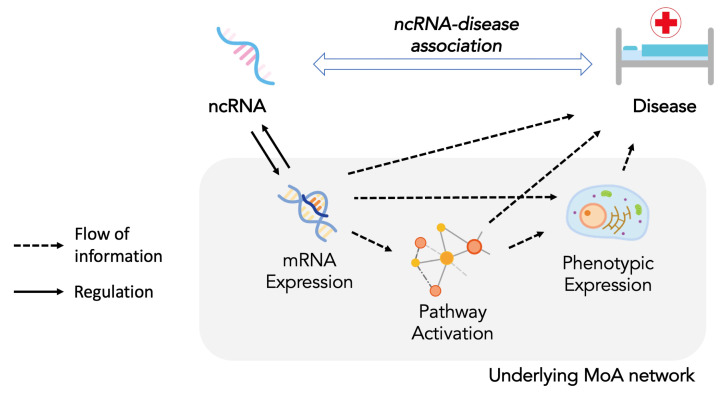
The MoA network of ncDA. NcRNA, regulating gene expression, alters the biological pathway and induces change of cellular phenotype. Aggregation of cellular phenotypes results in disruption of homeostasis and leads to a disease state.

NcRNA-disease association can be studied with the MoA concept analogous to the drug-disease associations. We refer to the underlying network between ncRNA and disease as Mode of Action (MoA) network (Figure 1). Through this perspective, we can reflect the biological roles and effects of ncRNAs. For example, regulation of gene expression by ncRNAs, which leads to alteration of biological pathway activation and cellular or clinical phenotypes, can be modeled in the MoA network.

To the best of our knowledge, there are few methods that integrate all of the entities mentioned as nodes of the MoA network to predict ncDA. However, we were able to survey several studies covering multiple entities, and they can be categorized in terms of the coverage of the network. We summarize those methods and studies in the following sections.

## 5. Computational Methodologies for Modeling the MoA Network

**Figure 2:**
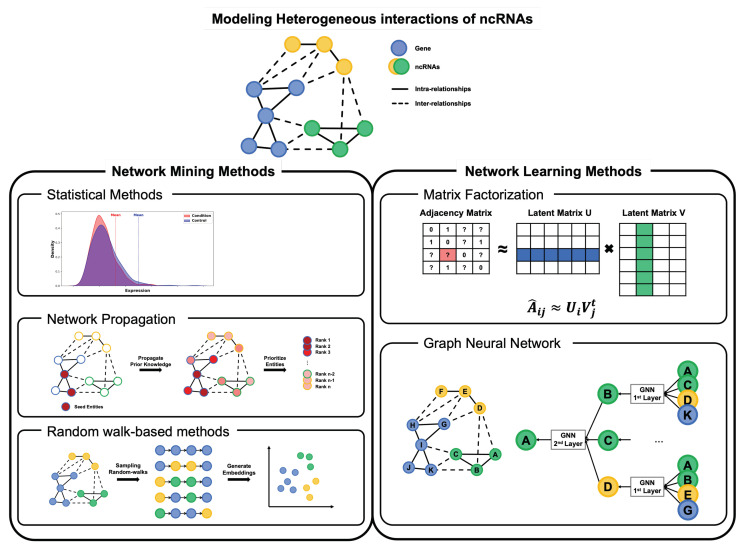
Two methodologies for investigating the relationship between ncRNA and other biological entities; network mining methods (statistical methods, network propagation, random walk-based methods) and deep learning methods (matrix factorization, graph neural network).

To infer potential ncDAs with the MoA network framework, it is critical to take advantage of valid associations between ncRNAs and other omics data. In this section, we introduce computational methodologies used to analyze the MoA network (Figure 2).

### 5.1. Methods for Mining on the MoA Network

#### 5.1.1. Statistical Methods

To construct ncRNA-disease associations (ncDAs), disease-specific ncRNAs should be identified. The most fundamental approach to identify those ncRNAs is differential expression (DE) analysis based on statistical methods. Expression profiles are measured from samples in different conditions, typically control and treated conditions. With these expression profiles as input, DE analysis tools determine genes or ncRNAs that show significantly different expression values between the conditions. DE ncRNAs can be directly used to connect potential ncRNA-disease associations. There exist various DEG tools such as limma-voom [52], DESeq2 [53], edgeR [54], ballgown [55], EBSeq [56], SAMSeq [57] and NOISeq [58].

A major limitation of DE statistical approaches is that DE ncRNAs and DEGs can hardly consider complex biological interactions. An interesting approach is to use DE analysis in two steps. Xin et al. [59] reported miRNAs for breast cancer antiestrogen resistance in a two step DE analysis where DE miRNAs were detected first and then only DEGs that are targeted by those DE miRNAs were used for identifying miRNAs for breast cancer antiestrogen resistance. In the predictive modeling or analysis, the major challenge is to balance false positives and false negatives. The success of this approach is to focus on reducing false positives in the two-step DE analysis. However, the weakness of this approach is then low sensitivity induced by stringent application of DE analysis twice.

#### 5.1.2. Network Propagation

Network propagation is a method used on biological networks for integrating and amplifying genetic signals from individual genes to their neighbors [60,61]. Specifically, given a biological network and a set of disease-associated genes, the genes are mapped to the network, and their effects are propagated to neighboring genes iteratively. After convergence, the algorithm returns a list of genes ranked in terms of disease relevance, and candidate disease-associated genes are unveiled through higher ranks. Generally, known disease-associated genes or DEGs are used as seed genes for initializing propagation and represented as $p_{0}$. Then, propagated information on genes at each iteration step *t*, $p_{t}$, can be calculated as follows:

$$p_{t+1}=Wp_{t}$$

where the normalized adjacency matrix of the network is *W*. Converged information on genes, $p_{\infty }$, can be analytically solved without simulating all the propagations. If ncRNAs are incorporated in the network, they are connected to their related genes in the network. Genes and/or ncRNAs that are already known to be related with a certain disease are used as seeds for network propagation. As a result of the network propagation, ncRNAs are ranked and ncRNAs with high rankings are regarded to be associated with the disease.

A variation of network propagation, widely known as Randomwalk with Restart (RWR) [62], added the idea of restart which leads to the calculation of proximity of entities from the initial seed genes [63]. It applied a restart probability α, which forces the propagation to always restart at the seed gene set $p_{0}$. RWR with the restart probability is computed by following equation:

$$p_{t+1}=\left(1-\alpha \right)Wp_{t}+\alpha p_{0}$$

#### 5.1.3. Random Walk-Based Methods

Random walk-based methods are utilized for creating node embedding vectors through random walk on the MoA network given as input. Random walks containing local structure information can be generated and summarized into a single node embedding vector by the skip-gram model [64,65]. For each entity $e_{i}$ on the random walks, the skip-gram model predicts contiguous entities $e_{j}$ included in the window size *w* by maximizing the objective function below.

$$L=\prod_{i=1}^{T}\;\prod_{j\neq i,\;j=i-w}^{i+w}\;p\left(e_{j}|e_{i})\right|$$

As a result, all the entities are mapped into the Euclidean space preserving their relative positions on the MoA network. Thus, ncRNAs and disease entities are represented in vectors considering the topology of the MoA network, which can be fed into downstream tasks such as link prediction to infer ncDAs.

### 5.2. Methods for Learning on the MoA Network

#### 5.2.1. Matrix Factorization

Matrix factorization is a method for decomposing a matrix into latent matrices that has been successful in user recommender systems [66]. Since ncRNA-disease associations can be represented as a matrix, predicting potential ncDAs can be formulated as matrix factorization. Given a matrix $X\in R^{n\times m}$, which represents the known ncRNA-disease associations between *n* ncRNAs and *m* diseases, the goal of matrix factorization is to approximate *X* by a product of a series of latent matrices by minimizing the objective function below.

$$L=∥X-UV^{⊤}∥$$

where $U\in R^{n\times k}$, $V\in R^{m\times k}$ are the latent matrices and *k* is the number of latent features. NcDAs are quantified in the matrix as one-hot or probability of positive association. Depending on the number of entity types composing the MoA network, more than two latent matrices are included in the decomposition formula representing associations with other entities such as mRNAs. The original ncRNA-disease association matrix can be reconstructed using the trained latent matrices, and the missing values are also completed through this process. The reconstructed output matrix can be understood as a predicted ncDA probability matrix.

#### 5.2.2. Graph Neural Networks

Graph neural network (GNN) is a deep learning-based method that directly incorporates a graph structure into the neural network and generates node embedding vectors [67]. Compared with random walk-based methods, GNN generates node embedding vectors through end-to-end learning that optimizes an objective function for the ultimate goal of ncDAs prediction.

Message passing is the most fundamental representation update scheme of GNN. It is divided into two steps: aggregation and update. The representation of a node *v* at *t*-th iteration is denoted as $R_{v}^{t}$. At (t+1)-th iteration, a message vector $M_{v}^{t+1}$ is created by aggregating messages of neighbor nodes’ representation at *t*-th iteration. Then, $R_{v}^{t+1}$ is updated with the message vector and the previous representation $R_{v}^{t}$. To summarize, it goes as follows:

$$\begin{array}{cc} M_{v}^{t+1} & =\sum_{w\in N(v)}A_{t}\left(R_{v}^{t},R_{w}^{t}\right)\\ R_{v}^{t+1} & =U_{t}\left(R_{v}^{t},M_{v}^{t+1}\right) \end{array}$$

where message aggregation and update functions are $A_{t}$ and $U_{t}$, and the list of neighbor nodes of *v* is annotated as N(v). Through this process, embedding vectors of the entities in the MoA network are computed so that local structure information of the network can be encoded in the embedding vectors. The resulting embedding vectors of GNN models can be directly used for ncDA prediction in an end-to-end manner.

## 6. Computational ncRNA-Disease Association Studies

The concept of the MoA network is now becoming a new trend in computational ncRNA-disease association prediction. Recently, several approaches have been proposed for leveraging various levels of biology in understanding diseases and ncRNAs’ roles in them. Some studies also report the improvement in prediction performance when integration of multiple level data is performed. In this section, studies covering various types of interaction in the MoA network are introduced. These integrative models can be split into two categories based on their coverage of the MoA network; ncRNA–mRNA–Disease integrative studies and ncRNA–mRNA–Pathway/Phenotype–Disease integrative studies. The following sections are organized into two sub-categories based on their base approaches: network mining and network learning methods. Network mining methods leverage network propagation or walk-based methods while network learning methods more aggressively utilize machine learning methods, from random forest to deep learning.

Before examining the MoA-network integrated studies, we briefly introduce ncDA studies that infer novel associations from known ncDA data only, without the consideration of other biological level entities.

In the year 2008, the analysis of Lu et al. [68] reported important network patterns of miRNA-disease associations. This result led to the proposal of various pioneering computational ncDA prediction algorithms based on a direct association network of diseases and ncRNAs. RWRMDA [69] was among the first studies to apply RWR algorithm for mining the global miRNA interaction network, and MIDP [70] used a random walk algorithm in mining the similarity network of miRNA and disease for ncDA prediction, while Yang et al. [71] performed a resource-allocation-based propagation algorithm on lncRNA-disease bipartite network for predicting lncDAs. IMCMDA [72] used an inductive matrix factorization method to infer the missing miRNA–disease association based on the known associations, miRNA similarity and disease similarity. Leveraging the ceRNA hypothesis Section 2.4, HGLDA [73] constructed a disease–miRNA–lncRNA network, which was passed on to a hypergeometric distribution-based model for lncDA prediction. Other tools for mining ncDAs from ncRNA-disease bipartite graph [74,75,76,77,78,79] are organized in Table 2.

With the rise of deep learning technology, numerous models have been proposed for learning the ncRNA-disease associations through neural networks, especially with GNN. Xuan et al. [80] leveraged graph convolutional network and convolutional neural network for predicting ncDA. GCNCDA [81] also leveraged Graph Convolutional Network (GCN) based on circRNA and disease similarity networks for circDA prediction. Recently, Sheng et al. proposed a multi-channel graph attention autoencoder named MGATE [82] for lncDA prediction. Additionally, a pioneering graph attention network model GTGenie [83] integrated ncRNA-disease similarity network with text-based relation representation based on BioBERT [84], a language model pretrained on large-scale biomedical corpora. The inputs, outputs reported ncDA prediction performances, and other detailed information on the representative tools of this section are provided in Table 3. Additional learning-based ncDA prediction tools [85,86,87,88] are also organized in Table 2.

### 6.1. ncRNA-mRNA-Disease Network

Many studies aimed to identify disease-associated ncRNAs through ncRNA–mRNA integrated analysis. For example, Xin et al. [59] applied a two-step analysis approach to discover and investigate the role of miRNAs in resistance to the drug fulvestrant. They predicted miRNAs and target mRNA transcripts that are relevant to fulvestrant resistance, and further performed pathway analysis which showed that these miRNAs regulate cancer-related signal cascades. We now summarize a list of studies that integrated not only ncRNA, but also mRNA information in the MoA network to find possible possible biomarkers of specific condition or disease-of-interest.

#### 6.1.1. Mining Based Studies

Various graph mining techniques have been actively applied to discover novel relationships from ncRNA–mRNA–disease tripartite networks. Most tools mine relationships from general template networks, while few approaches attempt to utilize target network information above miRNA–mRNA expression signatures.

Going beyond DE miRNA–DE mRNA coexpression analysis based on WGCNA [116], several tools have leveraged the ncRNA–mRNA target network for molecular interaction-level network mining. MIMRDA [110] incorporated miRNA-target pair information with DE miRNAs and DE mRNAs to predict miDAs (miRNA–disease associations). In order to identify key miRNAs of a given disease, the global probability value for each DE miRNA was computed from the significance level and result of over-representation analysis.

Several models have adopted different mining methodologies after building a tripartite network of ncRNA–mRNA–disease. RWRMTN [101], a Cytoscape app for novel miDA prediction, adopted RWR modified for heterogeneous network to prioritize miRNAs that are associated with query disease. Similarly, for discovering novel lncDAs, TPGLDA [92] used network propagation on bipartite/tripartite network and MHRWR [100] employed a random walk with restart-based algorithm.

Apart from RWR-based algorithms, several path-based algorithms have been proposed for mining the heterogeneous network of ncRNA–mRNA–disease. To consider the heterogeneity of the network, MDPBMP [111] leveraged the meta-path concept into a miRNA–disease–gene network. After the selection and application of seven meta-paths, embedding vectors of miRNA and disease were generated and utilized for miDA prediction.

As more and more evidence of ceRNA interactions accumulated, a number of studies leveraging this ncRNA interaction network for ncDA prediction have emerged. Along with co-expression analysis of lncRNA–miRNA–mRNAs, the ceRNA network was integrated for network-based analysis of ncRNAs related to gastric cancer [96], schizophrenia [112], and coronary artery disease [117]. Another component of the ceRNA hypothesis, circRNA, has been also investigated based on its regulatory network with other transcripts. Lu et al. [99] constructed a circRNA–miRNA–mRNA regulatory network based on RNA-seq data for discovering novel Hantaan virus infection associated circRNAs based on network analysis approaches. SDNE-MDA [104] integrates not only the ceRNA interaction network, but also the drug–target interaction network for prediction of miDAs. Attribute information of miRNA and disease was extracted from similarity networks of sequence and disease hierarchy each, and behavior information from a drug–protein–transcript interaction network. MiDA prediction was performed by feeding the concatenated attribute and behavioral information into the Convolutional Neural Network.

RWR algorithm has been also used for mining the ceRNA–mRNA integrated network for discovering novel ncDAs, mainly through the construction of ncRNA and disease similarity networks. A pioneering study by Song et al. [89] in 2016 constructed a lncRNA–mRNA network through integrating miRNA target information and gene expression profiles for blending in the ceRNA hypothesis. Then, the RWR algorithm was applied for discovering potential cardiac hypertrophy-associated lncRNAs. To make more use of the lncRNA–miRNA regulatory network, LRWRHLDA [113] constructed four novel similarity networks of lncRNA, disease, miRNA and gene. A Laplacian normalized RWR was then performed on the constructed heterogeneous network for prioritization of disease-related lncRNAs.

#### 6.1.2. Learning Based Studies

Leveraging the power of machine learning in extracting patterns from high-dimensional data through supervised settings, several tools have been developed to integrate ncDAs from ncRNA–mRNA expression profiles.

miRModuleNet [114] is a tool which integrates miRNAs and mRNAs expression profile through application of novel G-S-M approach. The pipeline of three components, Grouping component, Scoring component and Modeling component, is performed iteratively for prioritization of miRNAs. First, G-component maps multiple genes per one miRNA as ‘targets’, and these groups are then passed on to M-component, a random forest algorithm-based ranking function, along with S-component for scoring the feature importance of given miRNA group.

LGDLDA [105] predicted lncDAs through a neural network neighborhood information aggregation-based supervised learning framework on similarity matrix of lncRNA–gene–disease network. First, similarity networks for each lncRNA, gene, disease were constructed using interaction networks including lncRNA–miRNA and disease–miRNA networks. After neural network neighborhood information aggregation, LGDLDA predicts the lncRNA–disease association for accurately predicting the existing lncDAs and also discovering novel associations.

Cr-NMF [106] is a co-regularized non-negative matrix factorization method that integrates the lncRNA expression, gene interaction network, gene–lncRNA associations, and disease–gene associations. The disease–lncRNA association is factorized by this method and other information, such as gene interactions, gene–lncRNA associations and disease–gene associations, are integrated as a regularization term.

DRAMA [115], a GCN-based model proposed by He et al. predicts circDA utilizing ceRNA interaction information. After initializing similarity matrices of circRNA, miRNA, mRNA and disease from various sources with Principal Component Analysis, GCN is applied for aggregating local neighbor information of nodes. After training the neural network, a triple entity correlation measure is applied for extracting mRNA–miRNA–circRNA axis candidates related to a given disease.

A pioneering model MOGONET [47] utilized artificial intelligence technology for integrative miDA discovery through applying GCN and view correlation discovery network (VCDN) on multi-omics data. First, from mRNA expression, DNA methylation and miRNA expression profiles of multiple samples, sample similarity networks were constructed using GCN. Then, a GCN-constructed cross-omics tensor of mRNA, methylation and miRNA was passed on to the VCDN [118], originally developed for human action recognition tasks. VCDN was applied to learn the latent sample space by a multi-view approach based on a generative loop of a generator–discriminator neural network framework. It is worth noting that MOGONET models trained with three types of omics achieved the best performance in biomedical data classification and biomarker discovery tasks, compared to the models trained with single type, demonstrating the effectiveness of multi-omics integration, which is a core concept of the MoA network.

Detailed information on representative models of ncRNA–mRNA–disease integrated studies, including their inputs, outputs and if available, ncDA prediction performances reported, are organized in Table 4.

### 6.2. ncRNA-mRNA-Pathway/Phenotype-Disease Network

To incorporate biological knowledge a priori to find ncDAs, several recent studies integrated pathway or phenotype information along with mRNA expression data. Those tools cover the MoA network much more, bridging the gap between molecular and clinical information. There are a few mining-based approaches, but there are not many studies yet that propose a learning framework that takes pathway or phenotype information into account.

#### Mining Based Studies

A few studies analyze the correlation of miRNA expression with the genes included in a certain pathway. Wilk et al. [93] discovered miRNAs associated with disturbed pathways in cancer through a pathway activation score (PAS), calculated by incorporating pathway information with mRNA expression data using dimensionality reduction technique Isomap. Additionally, Tian et al. [90] identified *MYC* gene and its regulator miRNA hsa-miR-423-5p as a hub nodes and potential biomarkers in nasopharyngeal carcinoma (NPC), which was revealed by integrated analysis of miRNA-mRNA-pathway network. Based on ceRNAs hypothesis, Wang et al. [107] constructed a Myasthenia gravis-specific lncRNA–SNPs meditated by ceRNA regulatory networks based on risk pathways, proposing the lncRNA–SNP–mRNA–pathway axis. Zhang et al. [108] incorporated pathway information while performing WGCNA to construct a mRNA–lncRNA–pathway coexpression network and selected hub lncRNAs and mRNAs as diagnostic markers of pediatric sepsis. A pipeline called ImmLnc [102], developed by Li et al., was used to identify immune related lncRNA biomarkers in cancer, using a novel scoring system called lncRES (lncRNA-immune related pathway relation score). Qi et al. [97] suggested a method to extract significant lncRNA by dysfunctional pathway crosstalk in lung adenocarcinoma (LUAD). They selected LUAD-related pathways through network propagation-based analysis and used WGCNA to determine lncRNAs in co-expression relation with crosstalk genes of the selected pathways.

Meanwhile, phenotype information, which includes cancer molecular subtypes [109], pathological stage [94], or drug sensitivity [95,98], can help us analyze the ncDAs. Singh et al. [45] proposed a framework named DIABLO, which is a canonical correlation analysis-based tool. This tool can integrate miRNA–mRNA expression, methylation, protein and metabolite data considering disease phenotypes, e.g., cancer molecular subtypes, for biomarker discovery. Mens et al. [119] revealed miRNAs related to cardiometabolic risk factors and diseases by conducting an analysis of miRNA expression, single nucleotide polymorphism (SNP), methylation, and phenotype information (lipid and obesity-related traits, blood pressure, and so on). He et al. [120] discovered that lncRNA PVT1 is associated with NPCs through integrating phenotypes such as proliferation, apoptosis, and radio sensitivity with DE lncRNA. In addition, Xu et al. [91] designed a systematical lncRNA prioritization approach called LncNetP, which is based on cerna hypothesis and disease phenotype association assumptions. They predicted four candidate lncRNA genes (RHPN1-AS1, AC007389.1, LINC01116 and BMS1P20) that could serve as risk factors for diagnosis and prognosis. Gao et al. [103] constructed co-expression networks for lncRNA–mRNA, circRNA–mRNA, and miRNA–mRNA for endocrine therapy resistant breast cancer based on cellular phenotypes. Then, they established RNA crosstalk networks (lncRNA–miRNA–mRNA and circRNA–miRNA–mRNA) and predicted the functional roles of related ncRNAs.

The inputs, outputs and other detailed information on representative studies of this section are provided in Table 5.

Additionally, the studies introduced in the Section 6 are summarized in Table 2, based on their coverage of the MoA network and published year.

## 7. Discussion

In this survey, we introduced well-known ncRNAs and pivotal studies that revealed their association with diseases. The key to the process of linking ncRNA to disease is on how to incorporate biological relevance by integrating associations of genes and pathways. For this goal, our survey focuses on statistical and machine learning methods in the context of the mode of action (MoA) network. First, we elaborated the definitions of each type of ncRNAs, their biological roles, and evidence of their relationships with diseases. Second, we described an integrative point-of-view called the mode of action (MoA) network, which takes complex interactions of gene expression, biological pathways, and phenotypes into account. Third, major computational methodologies used for inferring ncDAs were introduced. Lastly, we summarized and categorized existing studies based on their coverage of the MoA network and whether the method discovers novel associations through mining or learning.

It is clear that ncRNAs are important entities serving critical regulatory roles in biological systems, in which its disturbance may lead to disease onsets. Many experimental studies aim to provide convincing evidence of the ncDAs, and computational studies leverage this known information to uncover novel and potential associations for further experimental studies. We summarized those studies in Table 2 in a temporal manner, and discovered two major trends. First, the network learning frameworks have been actively proposed after the year 2019, which correlates to the time when deep learning frameworks became actively adopted to the biological domain, especially as their applicability to networks became more reliable. Second, the MoA network integrative approaches, compared to direct association prediction methods, became more actively studied from 2020. Although previous studies have covered only a fraction of the MoA network, recent research considers not only direct relationships, but also mRNA expression data and/or pathway/phenotype information to bridge the gap between ncRNA and disease.

This trend is also showing promising results in other biological domains, including drug response prediction and patient stratification, where approaches that incorporate most of the entities in the MoA network already showed promising results. For example, the DrugCell [121] model integrates cancer mutational signals with Gene Ontology (GO) terms for interpretable drug response prediction and clinical outcome stratification. An interpretable neural network is constructed based on the semantics of the GO terms, and through training of 500,000 cell line-drug pairs, the model predicts drug response and simultaneously visualizes mechanisms related to response. Another pharmacogenomic framework named DRIM [122] integrates multi-omics data and pathway information for understanding the effect of drug treatment. Based on potential mediator genes selected from multi-omics data through tensor decomposition and autoencoder methods, the model analyzes time-series gene expression data upon drug treatment for identifying perturbed sub-pathways and regulation mechanisms.

Thanks to the development of ncRNA sequencing and deep learning technologies, we will have access to tons of high quality data and state-of-the-art analysis tools, which will help us better understand the biological roles of ncRNAs. Despite the lack of studies that formulate the ncDAs into a learning framework, integrating various types of information of the MoA network is a promising approach and will be a future direction for discovering ncDAs.

## Figures and Tables

**Table 1:** Summary of various ncDA databases.

Database	ncRNA Type	Description	URL
HMDD v3.2 [31]	miRNA	This database contains experimentally supported, manually curated evidence for the associations between human miRNAs and diseases.	https://www.cuilab.cn/hmdd, accessed on 26 August 2022
miR2Disease [32]	miRNA	This database is a manually curated database providing a comprehensive resource of miRNA deregulation in human diseases.	http://www.mir2disease.org/, accessed on 26 August 2022
dbDEMC [33]	miRNA	This database is an integrated database designed to retain and show differentially expressed miRNAs in cancers detected by high-throughput and low-throughput methods.	https://www.biosino.org/dbDEMC/index, accessed on 26 August 2022
miRCancer [34]	miRNA	This database provides a comprehensive collection of miRNA expression profiles from various human cancers.	http://mircancer.ecu.edu/, accessed on 26 August 2022
LncRNADisease v2.0 [35]	lncRNA circRNA	This database integrated comprehensive experimentally supported and predicted lncRNA- and circRNA-disease associations curated from manual literatures and other resources.	http://www.rnanut.net/lncrnadisease/index.php/home, accessed on 26 August 2022
Lnc2Cancer 3.0 [36]	lncRNA circRNA	This database is a manually curated database that provides comprehensive experimentally supported associations between lncRNA or circRNA and human cancer, with regulatory mechanisms, biological function, and clinical application.	http://bio-bigdata.hrbmu.edu.cn/lnc2cancer/, accessed on 26 August 2022
MNDR v3.1 [37]	miRNA lncRNA circRNA	This database integrated various kinds of mammalian ncDA through manual curation and prediction algorithms.	https://www.rna-society.org/mndr/home.html, accessed on 26 August 2022
CircRNADisease [38]	circRNA	This database contains a manually curated experimentally supported human circRNA-disease association.	http://cgga.org.cn:9091/circRNADisease/, accessed on 26 August 2022
CircR2Disease v2.0 [39]	circRNA	This database provides experimentally validated circRNA-disease association.	http://bioinfo.snnu.edu.cn/CircR2Disease_v2.0/, accessed on 26 August 2022
circAD [40]	circRNA	This database is a manually curated resource for dysregulated circRNAs in disease, with primer details for respective circRNAs and information about related genes.	https://clingen.igib.res.in/circad/, accessed on 26 August 2022
LncR2metasta [41]	lncRNA	This database is a manually curated database providing experimentally supported lncRNAs that are deregulated in cancer metastatic events, such as cancer cell invasion, proliferation and so on.	http://lncr2metasta.wchoda.com/, accessed on 26 August 2022
CircMine [42]	circRNA	This database provides comprehensive interactions between circRNAs and diseases with various physiological and pathological phenotypes, including drug resistance, disease stage, and so on.	http://www.biomedical-web.com/circmine/home, accessed on 26 August 2022

**Table 2:** Summary of computational ncRNA-disease association studies.

	Direct ncRNA-Disease Association		ncRNA-mRNA-Disease		ncRNA-mRNA-Pathway /Phenotype-Disease	
Year	Mining	Learning	Mining	Learning	Mining	Learning
∼ 2017	RWRMDA [69] RLSMDA [74] Yang et al. [71] HGLDA [73] MIDP [70] HGIMDA [75] IRWRLDA [76] PBMDA [77]		Song et al. [89]		Tian et al. [90] LncNetP [91]	
2018	ELLPMDA [78]		TPGLDA [92]		Wilk et al. [93] Zhou et al. [94] Xia et al. [95]	
2019		Xuan et al. [80]	Zhang et al. [96]		DIABLO [45] Qi et al. [97] Uhr et al. [98]	
2020		GCNCDA [81] Li et al. [85]	Lu et al. [99] MHRWR [100] RWRMTN [101]		ImmLnc [102] Gao et al. [103]	
2021	Nguyen et al. [79]	AEMDA [86] iCDA-CMG [87]	SDNE-MDA [104]	MOGONET [47] LGDLDA [105] Cr-NMF [106]	Wang et al. [107] Zhang et al. [108] Evangelista et al. [109]	
2022		MGATE [82] GTGenie [83] KGANCDA [88]	MIMRDA [110] MDPBMP [111] Sabaie et al. [112] LRWRHLDA [113]	miRModuleNet [114] DRAMA [115]		

**Table 3:** Detailed information of representative direct ncDA predictive tools. D: Disease, DA: Disease Association, $G_{i-j}$: association graph of *i* and *j*, $Hier_{i}$: hierarchy of *i*, $Sim_{func}$: functional similarity, $Sim_{sem}$: semantic similarity, AUROC: Area Under Receiver Operating Characteristic curve, AUPR: Area Under Precision Recall Curve, *N/A*: Not Available.

Tool	Year	Method	Software Language	Input	Output	Performance	
RWRMDA [69]	2012	RWR	*N/A*	known miDA, mi-mi $Sim_{func}$	predicted miDA	AUROC	0.8617
MIDP [70]	2015	RWR	*N/A*	known miDA, D-D $Sim_{sem}$	predicted miDA	AUROC	0.862
HGLDA [73]	2015	Statistical	*N/A*	known lncDA, $G_{D-mi}$, $G_{lnc-mi}$	predicted lncDA	AUROC	0.7621
IMCMDA [72]	2018	MF	Matlab	known miDA, $Hier_{D}$, mi-mi $Sim_{func}$	predicted miDA	AUROC	0.8034
GCNCDA [81]	2020	GNN	Matlab	known circDA, D-D $Sim_{sem}$	predicted circDA	AUROC Accuracy	0.9090 0.9278
Nguyen et al. [79]	2021	RWR	*N/A*	known miDA, $Hier_{D}$	predicted miDA	AUROC AUPR	0.9882 0.9066
MGATE [82]	2022	GNN	Python	known lncDA, $Hier_{D}$, $G_{lnc-mi}$, $G_{D-mi}$	predicted lncDA	AUROC AUPR	0.964 0.413
GTGenie [83]	2022	GNN	Python	known miDA, Text decription of ncDA, D-D $Sim_{sem}$, nc-nc $Sim_{sem}$	predicted ncDA	miDA AUROC lncDA AUROC	0.9755 0.9810

**Table 4:** Detailed information of representative ncDA predictive tools using ncRNA–mRNA–Disease network. D: Disease, DA: Disease Association, DE: Differentially Expressed $G_{i-j}$: association graph of *i* and *j*, $Hier_{i}$: hierarchy of *i*, AUROC: Area Under Receiver Operating Characteristic curve.

Tool	Year	Method	Software Language	Input	Output	Performance	
MOGONET [47]	2021	GNN	Python	Multi-omics profile	Predicted phenotype Rank of biomarkers	-	
MHRWR [100]	2021	RWR	Python	known lncDA, $Hier_{D}$, $G_{lnc-gene}$	Predicted lncDA	AUROC	0.9134
MIMRDA [110]	2022	Statistical	R	DE miRNA, DE mRNA, $G_{mi-gene}$	Rank of miRNAs	-	
MDPBMP [111]	2022	GNN	Python	known miDA, $G_{mi-gene}$, $G_{D-gene}$	Predicted miDA	AUROC	0.9214
miRModuleNet [114]	2022	Statistical	Python	known miDA, miRNA Exp, mRNA Exp, $G_{mi-gene}$	Predicted phenotype Rank of miRNA modules	-	
LGDLDA [105]	2021	GNN	Matlab	known lncDA, lncRNA expression, $Hier_{D}$, $G_{lnc-mi}$, $G_{lnc-gene}$, $G_{D-gene}$, $G_{D-mi}$	Predicted lncDA	AUROC	0.9352

**Table 5:** Detailed information of representative ncDA prediction tools using ncRNA–mRNA–Pathway/Phenotype–Disease network. Exp: Expression profile, PW: Pathway, $G_{i-j}$: association graph of *i* and *j*.

Tool	Year	Method	Software Language	Input	Output
Wilk et al. [93]	2018	Statistical	R	mRNA Exp, miRNA Exp, $G_{gene-PW}$	Disease-related miRNA-pathway pair
Xia et al. [95]	2018	Deep learning	Python	mRNA Exp, miRNA Exp, Protein abundance, Drug descriptors	Predicted drug response Gene, protein, miRNA biomarkers
DIABLO [45]	2019	Statistical	R	Multi-omics profiles	Predicted phenotype Rank of biomarkers
ImmLnc [102]	2020	Statistical	Web page	mRNA Exp, lncRNA Exp	Predicted phenotype Rank of lncRNAs

## Data Availability

Not applicable.

## Abbreviations

The following abbreviations are used in this manuscript:

ncRNA	non-coding RNA
miRNA	micro RNA
lncRNA	long non-coding RNA
circRNA	circular RNA
siRNA	small interfering RNA
piRNA	piwi-interacting RNA
ceRNA	competing endogenous RNA
ncDA	ncRNA-Disease association
miDA	miRNA-Disease association
lncDA	lncRNA-Disease association
circDA	circRNA-Disease association
MoA	Mode of Action
DE	Differentially Expressed
DEG	Differentially Expressed Gene
GNN	Graph Neural Network
RWR	Random Walk with Restart
NPC	Nasopharyngeal carcinoma
LUAD	Lung Adenocarcinoma
SNP	Single Nucleotide Polymorphism
GO	Gene Ontology
